# HIV Prevention Program Eligibility Among Adolescent Girls and Young Women — Namibia, 2019

**DOI:** 10.15585/mmwr.mm7045a2

**Published:** 2021-11-12

**Authors:** Nickolas T. Agathis, Francis B. Annor, Rachel Coomer, Jennifer Hegle, Pragna Patel, Norbert Forster, Gabrielle O’Malley, Alison L. Ensminger, Rahimisa Kamuingona, Helena Andjamba, Molisa Manyando, Greta M. Massetti

**Affiliations:** ^1^Epidemic Intelligence Service, CDC; ^2^Division of Violence Prevention, National Center for Injury Prevention and Control, CDC; ^3^Division of Global HIV and TB, Center for Global Health, CDC; ^4^International Training and Education Center for Health, Department of Global Health, University of Washington, Seattle, Washington; ^5^Ministry of Gender Equality, Poverty Eradication, and Social Welfare, Windhoek, Namibia; ^6^U.S. Agency for International Development, Washington, DC.

The U.S. President’s Emergency Plan for AIDS Relief (PEPFAR) relies on comprehensive and reliable population data to implement interventions to reduce HIV transmission in high-incidence areas among populations disproportionately affected by the HIV epidemic. Adolescent girls and young women in sub-Saharan Africa account for a disproportionate number of new HIV infections compared with their male peers (*1*). The DREAMS (Determined, Resilient, Empowered, AIDS-free, Mentored, and Safe) program includes multisectoral, layered interventions aimed at reducing factors that contribute to vulnerability to HIV infection among adolescent girls and young women in PEPFAR-supported sub-Saharan African countries (*1*). Namibia, a southern African country with a population of approximately 2.55 million among whom approximately 8% live with HIV infection, had their DREAMS program first implemented in 2017* (*2*,*3*). Data from the 2019 Namibia Violence Against Children and Youth Survey (VACS), the most recent and comprehensive nationally representative data source available to study the epidemiology of violence and other HIV risk factors, were used to estimate the percentage of adolescent girls and young women aged 13–24 years who would be eligible for DREAMS program services. The prevalence of individual DREAMS eligibility criteria, which comprise known age-specific risk factors associated with HIV acquisition, were estimated by age group. Among all adolescent girls and young women in Namibia, 62% were eligible for DREAMS based on meeting at least one criterion. Common eligibility criteria included adverse childhood experiences, specifically exposure to physical, emotional, and sexual violence and being an orphan;^†^ and high-risk behaviors, such as early alcohol use,^§^ recent heavy alcohol use,^¶^ and infrequent condom use.** Using VACS data to estimate the prevalence of HIV risk factors and identify adolescent girls and young women at elevated risk for HIV acquisition in countries like Namibia with high HIV-incidence can inform programs and policies aimed at improving the well-being of these adolescent girls and young women and help control the HIV epidemics in these countries.

In 2019, Namibia’s Ministry of Gender Equality, Poverty Eradication, and Social Welfare led the country’s first VACS in collaboration with CDC,^††^ the International Training and Education Center for Health at the University of Washington, and the Namibia Statistics Agency. The Namibia VACS was a cross-sectional, nationally representative household cluster survey of randomly selected noninstitutionalized adolescents aged 13–17 years and young women and men aged 18–24 years.^§§^ Local survey workers conducted face-to-face interviews with participants and inquired about lifetime experiences of physical, emotional, and sexual violence and other adverse childhood experiences; associated risk and protective factors; and related health outcomes and behaviors. For participants aged 13–17 years, informed consent and assent were obtained from a parent or guardian and the participant, respectively. Informed consent was directly obtained from participants aged ≥18 years and other nondependent participants.^¶¶^ Free, direct, and locally accessible referrals to social support services were offered to each participant, and response plans were created and implemented on a case-by-case basis.

Because DREAMS aims to prevent HIV infection among adolescent girls and young women, this analysis was limited to adolescent girls and young women who did not have an HIV infection or whose HIV status was unknown (i.e., did not know or refused to disclose their status and refused voluntary HIV testing at time of the survey); girls and young women living with HIV (based on self-report or voluntary HIV testing at time of the survey)*** and boys and young men who participated in the Namibia VACS were excluded. Participants were considered eligible for DREAMS if they met at least one DREAMS criterion for their age group, based on responses to the VACS questionnaire (Table 1). Nationally weighted prevalence of meeting at least one criterion, and two or more criteria, for DREAMS eligibility were estimated for adolescent girls and young women aged 13–14, 15–19, and 20–24 years. The weighted prevalence of individual DREAMS eligibility criteria for each age group were also estimated. All analyses were conducted using SAS (version 9.4; SAS Institute), accounting for the complex survey design. The Namibia VACS was reviewed and approved by Namibia’s Ministry of Health and Social Services research ethics committee and the CDC Institutional Review Board.^†††^

**TABLE 1:** DREAMS* eligibility criteria for adolescent girls and young women and corresponding Violence Against Children and Youth Survey indicators or questionnaire items — Namibia, 2019

Age group/Criteria	Survey indicators
**13–14 yrs**	
Ever had sex	Ever had vaginal, anal, or oral sexual intercourse
History of pregnancy	Ever been pregnant
Lifetime experience of sexual violence	Ever experienced sexual violence in lifetime
Experience of physical or emotional violence (within last year)	Experienced physical violence or emotional violence in the previous 12 mos
Early alcohol use	Ever drank alcohol (more than a few sips)
Out of school	Not currently attending school
Orphanhood	One or more biologic parents deceased^†^
**15–19 yrs**	
Multiple sexual partners (in last yr)	Had >1 sexual partner in previous 12 months
History of pregnancy	Ever been pregnant
STI	Ever received a diagnosis of an STI or had a genital sore or ulcer
Infrequent or no condom use	Not always using a condom with ≥1 sexual partner in the past 12 mos^§^
Transactional sex (including staying in a relationship for material or financial support)	Had sex with someone for material support or help in the past 12 mos
Lifetime experience of sexual violence	Ever experienced sexual violence in lifetime
Recent heavy alcohol use	Had ≥4 drinks of alcohol on one occasion in the past 30 days
Out of school	Not currently attending school
Orphanhood	One or both biologic parents deceased before the 18th birthday^†^
**20–24 yrs**	
Multiple sexual partners (in last yr)	Had >1 sexual partner in previous 12 mos
STI (diagnosed or treated)	Ever received a diagnosis of an STI or had a genital sore or ulcer
Infrequent or no condom use	Not always using a condom with ≥1 sexual partner in the past 12 mos^§^
Transactional sex (including staying in a relationship for material or financial support)	Had sex with someone for material support or help in the past 12 mos
Lifetime experience of sexual violence	Ever experienced sexual violence in lifetime
Recent heavy alcohol use	Had ≥4 drinks of alcohol on one occasion in the past 30 days

**Abbreviations:** DREAMS = Determined, Resilient, Empowered, AIDS-free, Mentored, and SAFE; STI = sexually transmitted infection.

* Saul J, Bachman G, Allen S, Toiv NF, Cooney C, Beamon T. The DREAMS core package of interventions: a comprehensive approach to preventing HIV among adolescent girls and young women. PLoS One, 2018;13:e0208167.

^†^ Orphanhood defined as having one or both parents deceased before the 18th birthday per UNICEF.

^§^ Excludes adolescent girls and young women who reported being married or living with someone as being married and only having one sexual partner in past 12 months.

Overall, 4,211 girls and young women (89% response rate) completed the Namibia VACS. Among participating girls and young women, 175 (4.2%) had known HIV infection and were excluded from analysis. Among the 4,036 adolescent girls and young women without known HIV infection, 18%, 42%, and 40% were aged 13–14, 15–19, and 20–24 years, respectively.

Among all adolescent girls and young women in Namibia aged 13–24 years eligible for the Namibia VACS and without known HIV infection, 62% met at least one DREAMS criterion or risk factor, and 26% met two or more criteria (Figure). The highest prevalence of having at least one criterion was observed among adolescent girls aged 13–14 years (71%), followed by young women aged 20–24 years (63%). Even among the group with the lowest prevalence (aged 15–19 years), 57% had at least one DREAMS criterion. In addition, 28% of those aged 13–14 years, 28% of those aged 15–19 years, and 23% of those aged 20–24 years met two or more criteria.

**FIGURE:**
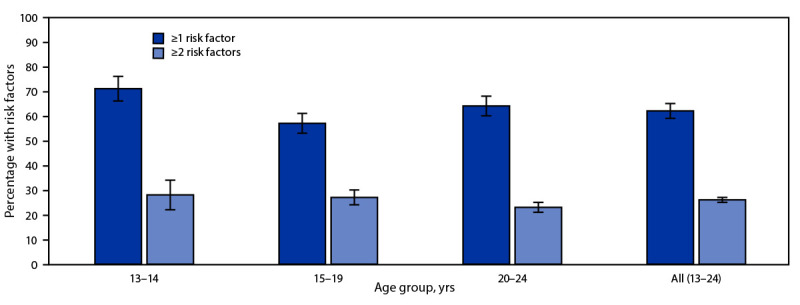
Prevalence* of having one or more or two or more risk factors for HIV infection^†^ among adolescent girls and young women, by age group — Namibia, 2019 **Abbreviation:** DREAMS = Determined, Resilient, Empowered, AIDS-free, Mentored, and Safe. * With 95% CIs, shown by error bars; all results were weighted to account for the survey design. ^†^ Presence of one or more HIV risk factors indicates eligibility for DREAMS programming (Saul J, Bachman G, Allen S, Toiv NF, Cooney C, Beamon T. The DREAMS core package of interventions: a comprehensive approach to preventing HIV among adolescent girls and young women. PLoS One, 2018;13:e0208167.

Among adolescent girls in Namibia aged 13–14 years, common DREAMS eligibility criteria that were met included experiencing physical or emotional violence in the past 12 months (50%),^§§§^ experiencing early alcohol use (21%), and having been orphaned (19%) (Table 2). Among girls and young women aged 15–19 years, common criteria included having been orphaned (23%), experiencing lifetime sexual violence (19%), and being out of school (18%). Among young women aged 20–24 years, common criteria included infrequent condom use in the past 12 months (39%), ever experiencing sexual violence (26%), and recent heavy alcohol use (18%).

**TABLE 2:** Prevalence* of risk factors for HIV consistent with DREAMS^†^ eligibility criteria among adolescent girls and young women aged 13–24 years (N = 4,036), by age group — Namibia, 2019

HIV risk factor	Age group, yrs											
	13–14			15–19			20–24			Total (13–24)		
	No.	Weighted % (95% CI)	Pop. est.	No.	Weighted % (95% CI)	Pop. est.	No.	Weighted % (95% CI)	Pop. est.	No.	Weighted % (95% CI)	Pop. est.
Ever had sex	742	3.9 (2.4–5.5)	1,713	1,698	40.5 (35.9–45.1)^§^	37,514	1,539	90.2 (88.3–92.1)^§^	76,780	3,979	52.5 (49.9–55.0)	116,007
History of pregnancy	742	0.3 (0.1–0.6)	150	1,698	15.3 (12.4–18.2)	14,148	1,536	56.5 (51.6–61.4)^§^	48,034	3,976	28.2 (25.3–31.0)	62,331
Experience of sexual violence in lifetime	741	10.6 (5.2–16.0)	4,610	1,709	19.3 (15.3–23.4)	17,955	1,569	26.0 (22.5–29.5)	22,309	4,019	20.2 (17.9–22.4)	44,874
Experience of physical or emotional violence in past 12 mos	745	49.5 (42.5–56.5)	21,567	1,709	44.8 (40.5–49.0)^§^	41,538	1,571	25.9 (22.4–29.3)^§^	22,258	4,025	38.4 (34.9–41.9)	85,363
Ever drank alcohol	716	21.3 (16.1–26.4)	9,048	1,639	44.9 (40.5–49.4)^§^	40,089	1,499	56.2 (51.5–60.8)^§^	46,904	3,854	44.6 (40.9–48.4)	96,041
Out of school	745	2.7 (1.0–4.5)	1,196	1,710	18.0 (15.2–20.9)	16,758	1,571	63.6 (60.9–66.4)^§^	54,714	4,026	32.7 (30.1–35.3)	72,667
Orphanhood^¶^	733	18.8 (14.1–23.6)	7,982	1,655	22.9 (19.6–26.2)	20,583	1,482	31.8 (27.4–36.2)^§^	26,047	3,870	25.5 (22.5–28.5)	54,612
Multiple sexual partners in past 12 mos	740	0.1 (0–0.3)^§^	46	1,693	3.4 (1.6–5.2)	3,164	1,526	6.0 (4.2–7.8)	5,106	3,959	3.8 (2.8–4.7)	8,316
STI**	743	3.8 (0.6–7.0)^§^	1,656	1,708	3.5 (2.4–4.6)	3,249	1,569	7.1 (5.1–9.2)	6,140	4,020	5.0 (3.8–6.1)	11,045
Infrequent condom use in past 12 mos^††^	739	0.6 (0.2–0.9)^§^	246	1,689	15.9 (13.2–18.5)	14,633	1,514	39.4 (35.5–43.4)	33,036	3,942	21.9 (20.4–23.4)	47,914
Transactional sex in past 12 mos	742	0.1 (0–0.3)^§^	63	1,705	1.4 (0.4–2.3)	1,261	1,567	2.5 (1.3–3.6)	2,128	4,014	1.6 (0.9–2.2)	3,451
Recent heavy alcohol use^§§^	735	4.6 (1.2–7.9)^§^	1,973	1,671	9.6 (7.2–12.0)	8,756	1,528	18.2 (15.6–20.7)	15,350	3,934	11.9 (10.2–13.7)	2,6079
DREAMS eligible (≥1 HIV risk factor)	745	70.6 (65.2–76.0)	30,742	1,711	57.0 (53.1–60.8)	52,898	1,580	63.1 (59.0–67.2)	54,963	4,036	62.0 (59.4–64.6)	138,603
≥2 HIV risk factors	745	27.6 (21.3–33.9)	12,007	1,711	27.5 (24.5–30.4)	25,516	1,580	23.1 (20.9–25.3)	20,130	4,036	25.8 (24.5–27.1)	57,653

**Abbreviations:** DREAMS = Determined, Resilient, Empowered, AIDS-free, Mentored, and Safe; Pop. est. = population estimate; STI = sexually transmitted infection.

* All results were weighted to account for the survey design.

^†^ Saul J, Bachman G, Allen S, Toiv NF, Cooney C, Beamon T. The DREAMS core package of interventions: a comprehensive approach to preventing HIV among adolescent girls and young women. PLoS One, 2018;13:e0208167.

^§^ Factors that do not represent DREAMS eligibility criteria for that age group.

^¶^ Orphanhood defined as having one or both parents deceased before the 18th birthday per UNICEF.

** STI was defined as reporting having received a diagnosis of a STI or having a genital sore or ulcer in lifetime.

^††^ Infrequent condom use defined as reporting no or infrequent condom use with at least one sexual partner in the past 12 months and excludes adolescent girls and young women who reported being married or living with someone as being married and only having one sexual partner in past 12 months.

^§§^ Recent heavy alcohol use defined as having had four or more drinks of alcohol on at least one occasion in the past 30 days.

## Discussion

Many adolescent girls and young women in Namibia experience increased risk for HIV acquisition, and the majority are eligible for DREAMS programming to prevent HIV infection. The 2019 VACS also found that one quarter of adolescent girls and young women met more than one DREAMS criterion, indicating that many are affected by multiple HIV risk factors requiring multipronged prevention strategies. The DREAMS approach of implementing multiple interventions, such as HIV and violence prevention programming, postviolence care services, HIV testing, preexposure prophylaxis, parenting/caregiver support, and a combination of socioeconomic approaches, addresses the multiple needs of adolescent girls and young women at risk for acquiring HIV. This multipronged approach can, in turn, lead to reductions in HIV risk behaviors, exposure to violence, and HIV and violence-related outcomes (*1*).

Physical, emotional, and sexual violence and other adverse childhood experiences were common and contributed to DREAMS eligibility. Experiencing violence is directly associated with increased risk for HIV acquisition and poor outcomes along the HIV care continuum (*4*). It is also associated with risk-taking behaviors, which can also increase the risk for HIV acquisition, and health consequences, including mental health problems, substance use, maternal health problems, and chronic diseases, all of which complicate HIV management (*5*). Primary and secondary prevention of violence is an integral part of DREAMS programming through emphasizing social asset building and safe spaces, changing harmful gender norms through community mobilization, promoting parenting/caregiving programming, and providing postviolence care (*1*). Expansion of violence prevention programming and services, using technical packages such as INSPIRE (*5*), can complement DREAMS and reduce violence in communities.

Early or recent heavy alcohol use and infrequent condom use in the past 12 months contribute to risk and were also commonly reported. These behaviors, along with other DREAMS eligibility criteria that were relatively uncommon, including having multiple sexual partners and participating in transactional sex in the past 12 months, increase risk for HIV acquisition among adolescent girls and young women in sub-Saharan Africa (*6*–*8*). DREAMS and other HIV prevention programs can leverage data-driven efforts to target risk-reducing interventions, such as HIV testing and partner testing, initiation of preexposure prophylaxis, economic strengthening, and school-based sexuality education, for populations at highest risk for HIV acquisition (*1*).

The findings in this report are subject to at least five limitations. First, data are self-reported and subject to recall and social desirability biases that might underestimate the prevalence of risk factors and behaviors.^¶¶¶^ Second, sampling excluded certain vulnerable subpopulations, particularly those experiencing homelessness or are institutionalized, and thus, these findings are not generalizable to those populations. Third, VACS questions might not precisely reflect all DREAMS eligibility criteria, and results might underestimate DREAMS eligibility. Fourth, a small proportion of responses (<5% for any variable) were missing, unknown, or declined, and were excluded from the analysis. Prevalence calculations were weighted to adjust for these responses. Finally, the analysis did not stratify or account for possible regional differences within Namibia.

These findings have significant implications for HIV prevention programming in Namibia and other PEPFAR-supported countries. VACS can complement data sources, such as the Demographic and Health Surveys (*9*), the Population-based HIV Impact Assessment Surveys (*10*), and DREAMS program data to inform efforts to tailor DREAMS and other HIV prevention programs. Estimates of DREAMS program eligibility among adolescent girls and young women can guide resource planning and prioritization, offer a baseline estimate for monitoring and evaluation, and improve stakeholder engagement by emphasizing the confluence of factors that increase the risk for HIV among adolescent girls and young women. Specifically, in Namibia, VACS implementers, government ministries and civil society partners, including nongovernmental organizations implementing DREAMS, conducted intensive data-to-action workshops from July–August 2020. During these workshops, Namibia VACS data were examined and discussed and recommendations for adjusting and adapting programming and policies were made. The findings and recommendations from this workshop have informed the current drafting of a national action plan aimed to address violence against children in Namibia. Consequently, Namibia VACS data have helped guide efforts to expand and adapt DREAMS and violence prevention and response programming in the country. Lastly, other PEPFAR-supported countries that implement VACS in the future could consider using VACS to identify participants at high risk and link them to DREAMS and other HIV and violence prevention programming in real time. Using VACS data in these ways can inform programs and policies aimed at improving the well-being of adolescent girls and young women and help to control the HIV epidemic in high-incidence countries.

**Summary**

**What is already known about this topic?**

HIV disproportionately affects adolescent girls and young women in high-incidence sub-Saharan African countries. The DREAMS (Determined, Resilient, Empowered, AIDS-free, Mentored, and Safe) program, supported by the U.S. President’s Emergency Fund for AIDS Relief, aims to reduce HIV incidence within this population.

**What is added by this report?**

Namibia’s 2019 Violence Against Children and Youth Surveys found that 62% of girls and young women aged 13–24 years were eligible for DREAMS programming, having one or more risk factors associated with HIV acquisition.

**What are the implications for public health practice?**

Use of nationally representative data can inform programs and policies aimed to improve the well-being of adolescent girls and young women and help control the HIV epidemic in high-incidence countries.

## Footnotes

* DREAMS program was first implemented in 2017 in three regions (Khomas, Oshikoto and Zambezi) and expanded to include two more regions (Kavango East and Oshana) in 2020.

† Orphanhood defined as having one or both parents deceased before the 18th birthday per UNICEF.

§ Early alcohol use defined as ever drinking more than a few sips of alcohol among adolescents aged 13–14 years.

¶ Recent heavy alcohol use defined as having had four or more drinks of alcohol on one occasion in the past 30 days.

** Infrequent condom use defined as reporting no or infrequent condom use with at least one sexual partner in the past 12 months and excludes those who reported being married or living with someone as being married and only having one sexual partner in the past 12 months.

†† As part of the Together for Girls Partnership. https://www.togetherforgirls.org

§§ In Namibia, VACS was implemented nationwide across the country’s 14 regions, with oversampling in three regions where DREAMS had been implemented in 2017 (Khomas, Oshikoto, and Zambezi).

¶¶ Namibia VACS also directly obtained consent from participants who were aged ≥16 years and had a child, were married under civil law, or were a child head of household.

*** Namibia’s antiretroviral therapy guidelines recommend that, in most situations, only children aged ≥14 years can consent to HIV testing without parental or guardian consent.

††† 45 C.F.R. part 46; 21 C.F.R. part 56; https://www.cdc.gov/violenceprevention/childabuseandneglect/vacs/country-process.html

§§§ Among adolescent girls aged 13–14 years, 29% reported experiencing physical violence and 40% reported experiencing emotional violence in the past 12 months.

¶¶¶ Certain measures were implemented to maximize disclosure, help ensure confidentiality, and reduce biases related to self-reporting, including a split sample approach that required sampling females and males from different sampling units, private face-to-face interviews, and an informed consent process that assured participants of the confidentiality of their responses.
